# Antimicrobial Susceptibility of *Escherichia coli* Isolated from Fresh-Marketed Nile Tilapia (*Oreochromis niloticus*)

**DOI:** 10.1155/2014/756539

**Published:** 2014-04-07

**Authors:** Rafael dos Santos Rocha, Lana Oliveira Leite, Oscarina Viana de Sousa, Regine Helena Silva dos Fernandes Vieira

**Affiliations:** ^1^Laboratory of Environmental and Fish Microbiology, Marine Sciences Institute, Federal University of Ceará, Abolição Avenida, 3207, 60168-081 Fortaleza, CE, Brazil; ^2^Fisheries Engineering Department, Federal University of Ceará, Mister Hull Avenida, 60356-000 Fortaleza, CE, Brazil

## Abstract

The contamination of seafood by bacteria of fecal origin, especially *Escherichia coli*, is a widely documented sanitary problem. The objective of the present study was to isolate *E. coli* strains from the gills, muscle, and body surface of farmed Nile tilapias (*Oreochromis niloticus*) fresh-marketed in supermarkets in Fortaleza (Ceará, Brazil), to determine their susceptibility to antibiotics of different families (amikacin, gentamicin, imipenem, cephalothin, cefotaxime, ciprofloxacin, aztreonam, ampicillin, nalidixic acid, tetracycline, and sulfametoxazol-trimetoprim), and to determine the nature of resistance by plasmid curing. Forty-four strains (body surface = 25, gills = 15, muscle = 4) were isolated, all of which were susceptible to amikacin, aztreonam, cefotaxime, ciprofloxacin, gentamicin, and imipenem. Gill and body surface samples yielded 11 isolates resistant to ampicillin, tetracycline, and sulfametoxazol-trimetoprim, 4 of which of plasmidial nature. The multiple antibiotic resistance index was higher for strains isolated from body surface than from gills. The overall high antibiotic susceptibility of *E. coli* strains isolated from fresh-marketed tilapia was satisfactory, although the occasional finding of plasmidial resistance points to the need for close microbiological surveillance of the farming, handling, and marketing conditions of aquaculture products.

## 1. Introduction

The bacterium* Escherichia coli* is widely used as indicator of the bacteriological condition of food and environments due to its almost exclusively fecal origin [1]. The presence of* E. coli* in fresh-marketed seafood indicates recent contamination and is usually attributed to infected handlers or storage on contaminated ice [2].

The intensification of production and the consequent increase in stocking density have made fish farming more vulnerable to disease [3, 4]. The indiscriminate use of antibiotics to treat infections and promote growth has been shown to be inefficient in the long run and to put selective pressure on bacterial populations favoring the development of resistant strains potentially hazardous to public health [5–7].

In fact, bacterial strains resistant to different families of antibiotics have been isolated from environmental samples by a number of researchers [8–10]. Foodborne strains resistant to antibiotics pose a risk to consumers' health and favor the transference of the phenotype to humans through the food chain [11–13]. There are no reports of illnesses caused by* E. coli* in farmed fish, but resistant strains may be selected due to the presence of antibiotics in the culture environment, leading to the dissemination by mobile genetic elements of resistance to potentially pathogenic bacteria [14–17].

Due to the importance of tilapia farming in Northeastern Brazil, the aim of the present study was to (a) investigate the presence of* E. coli* in fresh-marketed Nile tilapia obtained from supermarkets in Fortaleza, Brazil, (b) establish the antibiotic susceptibility profile of* E. coli* strains isolated from Nile tilapia samples, (c) determine whether resistance was potentially chromosomal or plasmidial, and (d) determine the multiple antibiotic resistance index of strains isolated from gills, muscle, and body surface.

## 2. Materials and Methods

### 2.1. Sample Collection

Thirty-six specimens of Nile tilapia (*Oreochromis niloticus*) were collected from twelve supermarkets in Fortaleza (Ceará, Brazil). The specimens were wrapped individually in plastic film and transported in ice-cooled isothermal boxes to the Laboratory of Seafood and Environmental Microbiology of the Marine Sciences Institute (Federal University of Ceará) for immediate bacteriological analysis.

### 2.2. Isolation of* Escherichia coli*

The* E. coli* investigation followed the guidelines of the fourth edition of the Compendium of Methods for the Microbiological Examination of Foods released by the American Public Health Association [18]. Presumptive tests were performed separately for gills, muscle, and body surface. To sample the body surface, an area measuring 10 × 10 cm was stroked with a sterile cotton swab previously soaked in Difco brain heart infusion (BHI) broth and subsequently immersed in 9 mL 0.85% NaCl solution (Vetec) diluted serially to 10^−4^. To sample the gills, a 25 g aliquot was homogenized in 225 mL 0.85% NaCl solution, shaken in a magnetic stirrer for 30 min, and diluted serially to 10^−6^. To sample the muscle, a 25 g aliquot was ground and homogenized in 225 mL 0.85% NaCl solution and diluted serially to 10^−3^. A 1 mL aliquot was retrieved from each saline dilution and seeded with three repetitions in a test tube containing 10 mL lauryl sulfate tryptose (LST, Difco). The samples were then placed in a bacteriological incubator at 35°C for 48 hours. Aliquots from positive LST tubes were seeded in 4 mL tubes containing EC broth and incubated in a water bath at 45°C for 48 hours.* E. coli *was isolated using eosin-methylene blue agar plates (Difco), from which 3–5 colonies suspected of* E. coli *were selectedand submitted to IMViC testing. Colonies were considered to be* E. coli* when positive in the indole and methyl-red test, negative in the Voges-Proskauer and citrate test, and Gram-negative with short rods in the Gram staining test [16].

### 2.3. Antimicrobial Susceptibility

The antibiogram was done with the disk diffusion method [19] using Mueller-Hinton agar (Difco). The standard strain* E. coli *ATCC 25922 was used as positive control [18]. Initially, an emulsion of sample in saline solution was prepared by adjustment to the 0.5 McFarland turbidity standard, equivalent to 1 × 10^8^ CFU·mL^−1^ (CLSI 2010). The susceptibility of the* E. coli* strains was tested in relation to several families of antibiotics, including the aminoglycoside family: amikacin (AMI; 30 *μ*g) and gentamicin (GEN; 10 *μ*g); the carbapenem family: imipenem (IMP; 30 *μ*g); the cephalosporin family: cephalothin (CET; 30 *μ*g) and cefotaxime (CTX; 30 *μ*g); the fluoroquinolone family: ciprofloxacin (CIP; 5 *μ*g); the monobactam family: aztreonam (ATM; 30 *μ*g); the penicillin family: ampicillin (AMP; 30 *μ*g); the quinolone family: nalidixic acid (NAL; 30 *μ*g); the sulfonamide family: sulfametoxazol-trimetoprim (SUT; 25 *μ*g); and the tetracycline family: tetracycline (TC; 30 *μ*g). Using sterile tweezers, commercially available antibiotic disks (Laborclin) were placed individually on the surface of Mueller-Hinton agar. After 24 hours of incubation at 35°C, the strains were scored as “susceptible,” “intermediate,” or “resistant” to each antibiotic based on the measurement of the inhibition halo, as recommended by CLSI [20].

### 2.4. MAR Index

The multiple antibiotic resistance (MAR) index was determined for the total number of* E. coli* strains from each type of tissue sampled (gills, muscle, and body surface) using the formula *a/(b* · *c*), where *a* is the total resistance score of the strains, *b* is the total number of families of antibiotics tested, and *c* is the number of strains from each type of tissue sampled [21].

### 2.5. Plasmid Curing

Resistant* E. coli *strains were submitted to plasmid curing using acridine orange dye at 100 *μ*g·mL^−1^ (Sigma). Following exposure to the mutagen, the strains were rechallenged with the antibiotics to which they were initially resistant [22].

## 3. Results and Discussion

Forty-four of the isolates were confirmed to be* E. coli*, 25 (56.82%) of which were isolated from gills, 15 (34.09%) from the body surface, and 4 (9.09%) from muscle. Eleven* E. coli* strains isolated from gills and body surface were resistant to AMP, SUT, and TC, especially to last of these (gills *n* = 4; surface *n* = 3). On the other hand, strains isolated from muscle samples were susceptible to all the antibiotics tested (Table 1). All strains isolated from gills, muscle, and body surface were susceptible to AMI, ATM, CET, CTX, CIP, GEN, and IMP.

According to some authors, the gills have a more diversified microbiota, qualitatively and quantitatively, due to their direct contact with the water, especially in plankton feeders such as the Nile tilapia [23–26]. Mandal et al. [27] also detected* E. coli* in Nile tilapia muscle samples, but many authors believe that the muscle is a relatively innocuous tissue [28–30]. Nevertheless, the muscle may be contaminated during harvesting, a stressful process which often causes injury to the body surface, and/or during storage on contaminated ice [31, 32]. Molinari et al. [33] add that* E. coli *is commonly found in the gut of the tilapia; thus, to these authors, its presence in the muscle is an indication of poor handling practices.

Four resistance profiles were observed in this study: resistance to TC (*n* = 5), resistance to AMP (*n* = 4), resistance to SUT+TC (*n* = 1), and resistance to AMP+SUT+TC (*n* = 1) (Table 2). The profiles SUT+TC and AMP+SUT+TC involved more than one family of drugs and were therefore considered profiles of multiple antibiotic resistance [21]. The MAR indexes for strains isolated from body surface and gills were 0.037 and 0.026, respectively.

In a study by Jiao et al. [34] on the occurrence of* E. coli* in the gut of farmed Nile tilapia, the isolated strains were susceptible to AMI, ATM, CTX, and GEN, matching our own findings and supporting the notion that these antibiotics are little used in aquaculture.* E. coli* strains resistant to AMP, SUT, and TC were also reported by Ryu et al. [12]. Likewise, resistant strains of* E. coli* to TC were found by Wang et al. [35]. The authors suggest that improperly handled seafood is a critical reservoir for the dissemination of bacterial genes of multiple resistance. Acridine orange curing of the 11* E. coli* strains resistant to AMP, SUT, and TC revealed resistance to be plasmid-mediated in 4 cases and potentially chromosomal in 7.

According to Lamshöft et al. [36], sulfonamides are highly soluble in water and persistent in the environment. Thus, residues may be detected up to 10 days after administration, suggesting the possibility of detecting bacteria resistant to SUT for a relatively long period. Gao et al. [37] point out that tetracyclines and sulfonamides have a long history of use in aquaculture.

The presence of mobile genetic elements of resistance, especially plasmids and integrons, poses a risk to public health, as evidenced by Koo and Woo [38]. Not surprisingly, Tendencia and dela Peña [39] observed that the indiscriminate use of antibiotics in aquaculture has been paralleled by a significant increase in the number of reports of resistant bacteria isolated from aquaculture stock.

## 4. Conclusion

The overall high antibiotic susceptibility of* E. coli *strains isolated from fresh-marketed Nile tilapia was satisfactory, although the occasional finding of plasmid-mediated resistance points to the need for close microbiological surveillance of the farming, handling, and marketing conditions of aquaculture products. Nevertheless, it is necessary to note the origin of marketed fish in order to evaluate the potential risk to the consumer.

## Figures and Tables

**Table 1:** Number of susceptible, intermediate, and resistant strains of *Escherichia coli* isolated from the gills, muscle, and body surface of farmed Nile tilapias (*Oreochromis niloticus*) fresh-marketed in supermarkets in Fortaleza (Ceará, Brazil), 2012.

Tissue sampled	Antimicrobial agents tested										
	AMI	AMP	ATM	CET	CTX	CIP	GEN	IMP	NAL	SUT	TC
Gills											
S	15	12	15	11	15	15	15	15	14	14	11
I	0	1	0	4	0	0	0	0	1	0	0
R	0	2	0	0	0	0	0	0	0	1	4
Body surface											
S	25	20	25	23	25	25	25	25	24	24	21
I	0	2	0	2	0	0	0	0	1	0	1
R	0	3	0	0	0	0	0	0	0	1	3
Muscle											
S	4	4	4	4	4	4	4	4	4	4	4
I	0	0	0	0	0	0	0	0	0	0	0
R	0	0	0	0	0	0	0	0	0	0	0

S: susceptible; I: intermediate; R: resistant.

AMI: amikacin; GEN: gentamicin; IMP: imipenem; CET: cephalothin; CTX: cefotaxime; CIP: ciprofloxacin; ATM: aztreonam; AMP: ampicillin; NAL: nalidixic acid; SUT: sulfametoxazol-trimetoprim; TC: tetracycline.

**Table 2:** Antimicrobial resistance profiles of strains of *Escherichia coli* isolated from the gills, muscle, and body surface of farmed Nile tilapias (*Oreochromis niloticus*) fresh-marketed in supermarkets in Fortaleza (Ceará, Brazil), 2012.

Profiles	Tissue sampled	
	Body surface (*n* = 15)	Gills (*n* = 25)
AMP	1	3
TC	3	2
SUT + TC	—	1
AMP + SUT + TC	1	—

AMP: ampicillin; SUT: sulfametoxazol-trimetoprim; TC: tetracycline.
